# A Text Messaging–Enhanced Intervention for African American Patients With Heart Failure, Depression, and Anxiety (TXT COPE-HF): Protocol for a Pilot Feasibility Study

**DOI:** 10.2196/32550

**Published:** 2022-01-07

**Authors:** Judith Cornelius, Charlene Whitaker-Brown, Jaleesa Smoot, Sonia Hart, Zandria Lewis, Olivia Smith

**Affiliations:** 1 School of Nursing University of North Carolina at Charlotte Charlotte, NC United States; 2 Department of Public Health Sciences University of North Carolina at Charlotte Charlotte, NC United States; 3 Novant Health Charlotte, NC United States

**Keywords:** African American, heart failure, depression, anxiety, assessment, decision, administration, production, topical expert, integration, training and testing model, text messaging, SMS, minorities, behavior therapy

## Abstract

**Background:**

African Americans have a higher incidence rate of heart failure (HF) and an earlier age of HF onset compared to those of other racial and ethnic groups. Scientific literature suggests that by 2030, African Americans will have a 30% increased prevalence rate of HF coupled with depression. In addition to depression, anxiety is a predictor of worsening functional capacity, decreased quality of life, and increased hospital readmission rates. There is no consensus on the best way to treat patients with HF, depression, and anxiety. One promising type of treatment—cognitive behavioral therapy (CBT)—has been shown to significantly improve patients’ quality of life and treatment compliance, but CBT has not been used with SMS text messaging reminders to enhance the effect of reducing symptoms of depression and anxiety in racial and ethnic minority patients with HF.

**Objective:**

The objectives of our study are to (1) adapt and modify the Creating Opportunities for Personal Empowerment (COPE) curriculum for delivery to patients with HF by using an SMS text messaging component to improve depression and anxiety symptoms, (2) administer the adapted intervention to 10 patients to examine the feasibility and acceptability of the approach and modify it as needed, and (3) examine trends in depression and anxiety symptoms postintervention. We hypothesize that patients will show an improvement in depression scores and anxiety symptoms postintervention.

**Methods:**

The study will comprise a mixed methods approach. We will use the eight steps of the ADAPT-ITT (assessment, decision, administration, production, topical expert, integration, training, and testing) model to adapt the intervention. The first step in this feasibility study will involve assembling individuals from the target population (n=10) to discuss questions on a specific topic. In phase 2, we will examine the feasibility and acceptability of the enhanced SMS text messaging intervention (TXT COPE-HF [Texting With COPE for Patients With HF]) and its preliminary effects with 10 participants. The Beck Depression Inventory will be used to assess depression, the State-Trait Anxiety Inventory will be used to assess anxiety, and the Healthy Beliefs and Lifestyle Behavior surveys will be used to assess participants’ lifestyle beliefs and behavior changes. Changes will be compared from baseline to end point by using paired 2-tailed *t* tests. An exit focus group (n=10) will be held to examine facilitators and barriers to the SMS text messaging protocol.

**Results:**

The pilot feasibility study was funded by the Academy for Clinical Research and Scholarship. Institutional review board approval was obtained in April 2021. Data collection and analysis are expected to conclude by November 2021 and April 2022, respectively.

**Conclusions:**

The study results will add to the literature on the effectiveness of an SMS text messaging CBT-enhanced intervention in reducing depression and anxiety among African American patients with HF.

**International Registered Report Identifier (IRRID):**

PRR1-10.2196/32550

## Introduction

### Background

Improving the mental health needs of African American patients with heart failure (HF) fits within the public health mandate to reduce health disparities based on race and ethnicity [1-3]. Screening for depression in all patients with HF is a recent public health directive [1]. However, screening without evidence-based interventions for improving mental health is inadequate and does not result in positive outcomes. Cognitive behavioral therapy (CBT) is a form of psychological treatment that has shown effectiveness in treating a wide range of mental health conditions but has also shown mixed results with some populations when used alone [4]. An intervention that is based on CBT, targets patients with HF, and is designed to reduce depression and anxiety is vital, given the well-established link among depression, anxiety, and mortality [5]. The purpose of our exploratory feasibility study is to adapt an evidence-based CBT intervention by using SMS text messaging boosters to reduce depression and anxiety in African American patients with HF. This project will impact the mental health care of racial and ethnic minority patients with HF by reducing depression and anxiety, thereby improving the health outcomes of this population.

More than 5 million people in the United States experience congestive HF [1,2], and it is the most common diagnosis in hospitalized patients aged over 65 years [2]. Further, in 1 in 9 deaths, HF is a contributing factor to mortality. Moreover, in one study with adult patients, racial differences were noted among young patients with HF (aged <50 years) [6]. More specifically, African Americans have a 20 times higher incidence rate of HF compared to that of White Americans [2,6], and among patients aged younger than 75 years, African Americans have the highest incidence of HF and often have an earlier age of HF onset [6]. The American Heart Association estimates that by 2030, there will be a 30% increase (from 2012) in the prevalence of HF among African Americans [2].

Depression has been found to be an independent risk factor for mortality in patients with HF [1,5]. HF, depression, and anxiety share pathophysiological mechanisms. The prevalence of depression and anxiety in patients with HF is about 20% to 40%, which is 4% to 5% higher than that of the general population [2,5]. In addition to depression, anxiety is also a predictor of worsening functional capacity, decreased quality of life, increased hospital readmission rates, and increased mortality [1,5-7].

Structural racism is a fundamental driver of health disparities among racial and ethnic minority patients with HF [8]. African Americans sometimes experience more severe forms of mental health conditions due to unmet needs and other barriers, such as distrust in the health care system, which can cause many African Americans to not seek mental health treatment [7]. These factors underscore the urgent need for an adapted intervention that improves the mental health outcomes of this vulnerable population.

### Objectives

The proposed site for this study periodically screens its patients with HF for depression and anxiety during clinic visits and provides appropriate referrals to psychiatric services. However, the average wait time for the first psychiatric appointment remains high (116 hours), in part because 1 in 5 psychiatrists are not accepting new patients [9]. The provision of psychiatric care has been further impacted by the COVID-19 pandemic [9]. There is a lack of consensus on the best way to treat patients with HF, depression, and anxiety [3]. Some patients show improvement after taking medication, but medication does not have a significant benefit over placebos [5]. Psychotherapy may reduce depressive symptoms, but it does not affect disease outcomes [5]. One promising type of treatment—CBT—has been shown to significantly improve patients’ quality of life and treatment compliance [10].

### CBT Treatment

CBT is effective in treating insomnia, substance use disorder, schizophrenia, depression, anxiety, and anger in diverse patient populations, such as Hispanic and African American pregnant women, adolescents, and patients with HF^.^ [11,12]. However, few studies have examined the effectiveness of SMS text messaging reminders for patients with HF [13-16], and even fewer have examined the effectiveness of CBT for African American patients with HF [17]. The implementation of interventions with HF patients is not a one-size-fits-all solution, and gaps exist in understanding how CBT can be an effective treatment for depression and anxiety in racial and ethnic minority patients with HF. The question that remains unanswered is as follows: how does implementing a prescriptive, case-specific, ethnic- and cultural-driven SMS text messaging–enhanced intervention for racial and ethnic minority individuals with HF, depression, and anxiety improve health outcomes? Our proposed pilot study will begin to fill this gap by examining the effect of SMS text messaging–enhanced CBT for a racial and ethnic minority population—African Americans with HF—to improve their physical and mental health outcomes.

### Text Messaging–Based Interventions

SMS text messaging interventions can successfully modify adverse health behaviors. Compared to nonweb interventions, SMS text messaging interventions are effective in helping people with diabetes, cardiovascular disease, or prostate cancer achieve positive behavior change outcomes and improve their quality of life [13-17]. African Americans are more likely than other ethnic groups to use devices such as mobile phones, making interventions that involve SMS text messaging more likely to be acceptable to this population [13]*.* This type of platform is not only affordable but also effective. SMS text messaging is particularly suited for this target group because little effort is required to receive the text reminders, and such reminders can be accessed at times that are suitable for study participants [15-17]. This type of intervention can help to bridge the gap between the socioeconomic disparity related to vulnerable populations and the delivery of health care [16]. The outcomes of mobile phone SMS text messaging–enhanced interventions include increased exercise time, increased knowledge of nutrition, increased participation in health care, and weight loss maintenance, of which all are factors associated with maintaining a healthy lifestyle and reducing depression and anxiety among patients with HF. One study used SMS text messaging to improve HF self-management (eg, eating a low-salt diet, engaging in physical activity, and measuring daily weight) in an African American population [17].

A sufficient number of studies have shown the efficacy of CBT as an effective treatment strategy for mild to moderate depression when it is augmented with other delivery modalities [18,19]. Few studies have used SMS text messaging to reduce depression and anxiety among racial and ethnic minority patients with HF [16,17]. To our knowledge, our pilot study will be one of the few to use a CBT-based intervention with SMS text messaging boosters to alleviate depression and anxiety symptoms among African American patients with HF.

### Creating Opportunities for Personal Empowerment Intervention

The Creating Opportunities for Personal Empowerment (COPE) intervention consists of 7 brief, interactive CBT sessions that are evidence based, are readable at the sixth-grade level, and focus on empowering young adults to engage in healthy lifestyle behaviors, thereby improving mood and reducing depression and anxiety [11].

To our knowledge, we are the first to adapt the COPE intervention for use with SMS text messaging reminders to reinforce session content (Table 1). Our innovative study will result in a reproducible and scalable intervention for reducing depression and anxiety in ethnic minority patients with HF, thereby filling a needed gap in treatment for this population. We believe that this novel version of the COPE intervention, which is specifically designed for African American patients with HF (TXT COPE-HF [Texting With COPE for Patients With HF]), will have a synergistic impact on reducing the incidence of negative health care outcomes in this population.

**Table 1:** Examples of Creating Opportunities for Personal Empowerment sessions and proposed text message reinforcers to be sent (TXT COPE-HF [Texting With Creating Opportunities for Personal Empowerment for Patients With Heart Failure]).

Session	Content	Text message	Response examples
Session 1: Thinking, feeling, and behavior	Mindfulness activities	“What type of goal did you complete today?”	“Select & Send the # that matches your completed goal for today 1=Exercising 2=Journaling 3=Healthy Eating 4=Companionship 5=Other (please list)”
Session 2: Self-esteem and positive self-talk	Positive messages	“Did you remember to remind yourself that you are important?”	“Type Y for Yes and N for No”
Session 3: Stress and coping	Responses to stress	“Which coping technique did you practice today?”	“Select & Send the # that matches your completed stress/coping technique for today 10=Meditating 11=Deep breaths 12=Yoga 14=Other (please list)”
Session 4: Goal setting	How to set goals	“Did you complete a goal or action step today?”	“Type Y for Yes and N for No”
Session 5: Effective communication	Guided imagery	“What positive self-control strategy did you use today?”	“Select & Send the # that matches the 1 completed self-control strategy for today 15=Exercising 16=Healthy Eating 17=Companionship 18=Praying/Meditating 19=Other (please list)”
Session 6: Coping with stress	Coping skills	“What coping skill did you use today?”	“Select & Send the # that matches with 1 completed coping skill for today 22=Listening to music 23=Doing hobbies 24=Having quiet time 26=Reading 29=Other (please list)”
Session 7: Last session	Reinforcement of skills	“Did you practice a positive coping skill today?”	“Type Y for Yes and N for No”

### Specific Aims

The overall goal of our study is to improve health outcomes in African American patients with HF who experience mild depression and anxiety by adapting the existing COPE intervention for use with supplemental text messages. The specific aims for this pilot study are to (1) adapt and modify the COPE curriculum for delivery to patients with HF by using an SMS text messaging component to improve depression and anxiety symptoms, (2) administer the adapted intervention to 10 subjects to examine the feasibility and acceptability of the approach and modify it as needed, and (3) examine trends in depression and anxiety symptoms and conduct an exit focus group postintervention. We hypothesize that patients will show an improvement in depression scores and anxiety symptoms postintervention.

### Theoretical and Conceptual Framework

Guided by cognitive behavioral theory, we plan to adapt the COPE curriculum for young adults for use with supplemental SMS text messaging reminders to reach patients with HF and mild depression and anxiety (TXT COPE-HF). The COPE curriculum for young adults was adapted from other efficacious, cognitive-based, skill-building COPE programs directed at vulnerable populations (eg, racial and ethnic minority teens and pregnant women with depression) [11]. This intervention can be easily integrated into routine clinical care and support groups. On the basis of CBT, participants will be taught how to cognitively restructure their thinking when negative events and situations arise, as these tend to result in negative thoughts. Participants will learn how to restructure their thinking to create positive interpretations of negative events and situations, so that they feel better emotionally and behave in more healthy ways. Emphasis will be placed on how patterns of thinking impact behaviors and emotions (ie, the thinking, feeling, and behaving triangle). Goal setting for promoting healthy behaviors and problem-solving skills will be a part of the cognitive skill-building approach. We hypothesize that the TXT COPE-HF program will strengthen participants’ beliefs about and confidence in their ability to engage in healthy lifestyle behaviors and manage their negative emotions with text message booster reminders.

## Methods

### Research Design

This study will comprise a mixed methods approach. We will use the eight steps of the ADAPT-ITT (assessment, decision, administration, production, topical expert, integration, training, and testing) model to adapt the intervention (Table 2) [20]. The first step in this exploratory feasibility study will involve assembling individuals to discuss questions on a specific topic. We will gather data from focus groups during 2 phases by creating an environment that encourages participants to discuss their beliefs, perceptions, and points of view on the COPE for young adults curriculum and its applicability to African American patients with HF [11]. In phase 2, we will examine the feasibility and acceptability of the SMS text messaging component along with its preliminary effects. The research team has experience in working with racial and ethnic minority populations, and one member has experience in adapting a curriculum for text message delivery.

**Table 2:** Applying the ADAPT-ITT^a^ model to adapt the COPE^b^ curriculum for use with SMS text messaging reminders.

Study phases and ADAPT-ITT model steps		Description
**Phase 1**		
	Assessment	Conduct 2 focus groups with African American patients with heart failure (n=10)
	Decision	Decision made to adapt the COPE curriculum for use with SMS text messaging reminders
	Administration	Administer a theater test (trial) of text messages and analyze findings with 10 participants
	Production	Produce draft 1 of the adapted TXT COPE-HF^c^ program
	Topical expert	Consult with a topical expert about the adaptations
	Integration	Use feedback from the topical expert and create draft 2
**Phase 2**		
	Training	Train research team on the study’s outcomes and protocols
	Testing	Pretest the TXT COPE-HF program with 10 participants, conduct an exit focus group and analyze the findings, make modifications based on feedback, examine trends in depression and anxiety scores, disseminate findings, and modify the program as needed

^a^ADAPT-ITT: assessment, decision, administration, production, topical expert, integration, training, and testing.

^b^COPE: Creating Opportunities for Personal Empowerment.

^c^TXT COPE-HF: Texting With Creating Opportunities for Personal Empowerment for Patients With Heart Failure.

### Subjects

The sample sizes for phases 1 and 2 are based on our previous research on developing and pretesting text messages [21]. The participants in this study will be 30 African American patients with HF (20 patients in phase 1 and 10 patients in phase 2), and they will be recruited from a heart and vascular institute in North Carolina. For inclusion, phase 1 participants must be African American, be aged at least 21 years, understand what mobile phone SMS text messaging is, be diagnosed with HF, and speak and understand English. We will approach each African American patient who is treated at the heart institute about this study until we reach our desired sample size.

In phase 2, we will recruit African American patients with HF who meet the same inclusion criteria as those in phase 1 and have diagnoses of depression and anxiety. The participants in this group must have access to a mobile phone with SMS text messaging capabilities, so that they can access the TXT COPE-HF reminders.

An on-site staff member will answer questions from prospective participants, and the principal investigators’ contact information will be available on the flyer. A previously used scripted protocol will be used to inform prospective participants about the study. If they choose to participate, we will obtain consent on the first day of the study. The principal investigators will offer incentives for participation in the study, which we expect will help us attain and retain our sample. The compensation provided will be US $20 for the focus group participants (phase 1 and the exit focus group in phase 2) and US $20 for each completed TXT COPE-HF session in phase 2. The compensation amount is high because we aim to accommodate our population of patients with HF, who experience depression and anxiety and may tire easily.

### Setting

Focus groups will be conducted at the proposed site for the study. Focus group meetings will be held at the heart and vascular institute, and parking will be accessible for study participants. The two principal investigators have experience in moderating focus groups. Focus groups will be held in a conference room at the heart and vascular institute (ie, while adhering to social distancing guidelines) or be conducted via Zoom (Zoom Video Communications Inc; patient’s preference). The conference room offers access to computer equipment that the research team can use to display information from the COPE for young adults curriculum. The principal investigators will place audio recorders on a table in the middle of the room and use a flip chart to write down core ideas for adapting the COPE curriculum for SMS text messaging–enhanced delivery and modifying it for African American patients with HF.

### Instruments

The instruments and the reliability and validity values to be used in our pilot study are shown in Table 3.

**Table 3:** Instruments.

Study outcome	Instrument	Reliability values
Demographic characteristics (age, gender, income, level of education, and marital status)	Demographic survey	N/A^a^
Depression	Patient Health Questionnaire-9 (American Psychological Association, 2020 [22])	Cronbach *α* of .86-.89
Anxiety	State-Trait Anxiety Inventory (Spielbergere, 1989 [23])	Internal consistency coefficients of 0.86-0.95 and a test-retest reliability value of 0.65-0.75
Lifestyle beliefs	Healthy Lifestyle Beliefs Scale (Melynk et al 2014a [24], Melynk et al, 2014b [25])	Cronbach *α* of .91
Lifestyle behaviors	Health Lifestyle Behaviors Scale (Melynk et al, 2014b [25])	Cronbach *α* of .86
SMS text messaging	SMS text messaging plan	N/A

^a^N/A: not applicable.

### Procedure

In phase 1 of our project, we will train a graduate assistant and research assistant with expertise in SMS text messaging and research methods on the details of the study. We will develop an interview guide with probes that are consistent with the literature and are based on CBT. We will adapt the curriculum based on the ADAPT-ITT model that was used in our previous research [20,21]. After we receive institutional review board approval, we will begin recruitment. We do not anticipate any difficulty in obtaining the sample for this project. At 1 month before the initiation of the focus groups, we will post a sign-in sheet at the heart and vascular institute. This sheet will be used to announce our study and will include our names and contact information. Potential participants will be instructed to contact us if they are interested in participating in the study. When we are contacted by 10 potential participants (ie, 10 for each focus group session), we will schedule a meeting and invite these potential participants by telephone to attend this meeting. Participants will be required to attend 1 focus group session, which will last 1 to 1.5 hours. As participants gather for each focus group, they will review and complete the approved consent form and demographic survey. Before the start of the formal session, we will conduct an interactive activity to allow participants to become acquainted with each other and with us. After the welcoming of participants and a discussion of the rules for interaction, the formal focus group process will begin. Data from the focus group sessions will be used to adapt the COPE curriculum for SMS text messaging–enhanced delivery to African American patients with HF. Dosage (number of texts), text message content, and whether messages are being read and understood will be determined based on the focus group’s findings. In our previous study [16], we sent only 1 message per day, and participants were required to respond to the message with a 1-word response. We will use the TextMe app (TextMe Inc) to send the messages.

In phase 2, we will recruit patients with HF by using the same methods as those used to recruit the first group. We will administer the Patient Health Questionnaire-9 (PHQ-9), State-Trait Anxiety Inventory (STAI), demographic surveys, Healthy Lifestyle Behaviors Scale, and Healthy Lifestyle Beliefs Scale. We will invite 10 patients whose scores indicate mild depression (PHQ-9 scores: range 5-9) and anxiety (STAI scores: range 38-44) to participate in phase 2.

Phase 2 participants will receive daily SMS text messaging reminders (the number of reminders will be determined by the focus groups) after each session. After participants complete the seven sessions of the TXT COPE-HF curriculum, they will return for a follow-up exit focus group, during which we will readminister the surveys to examine trends in participants’ depression and anxiety scores, perceptions of healthy lifestyle behaviors and beliefs, and perceptions about the SMS text messaging reminders. The incentive provided will be US $20 for each SMS text messaging–enhanced session (n=7) completed and US $20 for the exit focus group session that will occur after session 7 (a total of US $160 per participant).

Each focus group will be audiotaped. The principal investigators will provide the audio recording equipment that they own and have used in previous studies. The research assistant will monitor the equipment while the focus group sessions are in progress. The researchers will use a single moderator, 1 interview guide, and observation notes from each session to ensure reliability. The research assistant will transcribe the audiotapes verbatim, and the transcripts will be compared to the original audio recordings and corrected as needed to ensure data completeness and accuracy.

### Data Analysis

The transcripts of the focus groups will be used as data for analysis. We will organize, store, and analyze the data by using NVivo qualitative analysis software (QSR International). The data analysis will begin with reading the transcribed interviews and listening to the audiotapes. The transcripts will be read multiple times by the research team to identify important phrases, paragraphs, sentences, and interactions. Analyses of the data will be conducted to categorize the responses according to a thematic topic guide (key components, group discussion responses, and delivery protocols). The principal investigators will read the data and compare their ideas to determine whether they have arrived at a consensus. Multiple meetings will be conducted until an agreement is met. Participants’ responses will guide the discussion on how we can adapt the components of the COPE curriculum for SMS text messaging–enhanced delivery (TXT COPE-HF).

As this is a pilot feasibility study that will have a small sample size, we will only be able to examine trends in depression scores, anxiety scores, and healthy lifestyle belief and behavior scores rather than examine differences in outcome effects. One principal investigator will oversee the data entry, management, and analysis processes. She will analyze demographic, feasibility, acceptability, and survey (PHQ-9, STAI, Healthy Lifestyle Beliefs Scale, Healthy Lifestyle Behaviors Scale, and the SMS text messaging evaluation plan) data by using descriptive statistics and 2-tailed *t* tests. The number of patients with HF who complete the SMS text messaging–enhanced intervention and their response rates in the session activities will be used as measures of feasibility. The number of participants who find the SMS text messaging–enhanced delivery method acceptable will be used as data for analyzing acceptability. Participants will be asked to comment on the overall text message delivery process by using a 4-point Likert scale ranging from 1 (strongly disagree) to 4 (strongly agree). We will enter quantitative data into SPSS version 28 (IBM Corporation) for analysis.

### Time Frame

The project can be completed within 12 months. We anticipate no problems in recruiting at least 30 subjects for the proposed study (Table 4).

**Table 4:** Protocol timeline.

Month	Activities	Responsible person	Outcome
1-2	Phase 1: obtain institutional review board approval, recruit participants for focus groups, and obtain consent	RA^a^, GA^b^, PIs^c^	Focus group participants provide consent for phase 1
3-5	Conduct focus groups, transcribe and analyze data, and share data with consultant	PIs, RA, and GA	Feedback from topical expert on findings from the focus groups that can be used in the adapted curriculum
6-8	Adapt curriculum for SMS text messaging–enhanced delivery, conduct trial, consult with consultant, and recruit and obtain consent for phase 2** **	PIs, RA, and GA	Participants provide consent for phase 2 of the study
9-11	Phase 2: collect data, conduct exit focus group, analyze data, and prepare for the dissemination of findings	Topical expert (with whom the findings of phase 2 will be shared)	Feasibility and acceptability data for the project and the examination of data for identifying trends
12	Disseminate findings and write a final report for submission	PIs, RA, and GA (topical expert will be acknowledged or be a coauthor)	Manuscripts submitted to journals for publication and to research conferences for presentation

^a^RA: research assistant.

^b^GA: graduate assistant.

^c^PI: principal investigator.

### Limitations

In this pilot feasibility study, we will have a sample of convenience, which may result in response bias. We are recruiting patients who own mobile phones or have access to one; hence, the findings may be different from those of patients who do not own a mobile phone. For future research, we may consider the inclusion of family members to assist patients who do not have access to a mobile phone or are not technologically adept. We also recognize that we are working with a population that may tire easily due to experiencing HF, depression, and anxiety. However, due to the enhanced SMS text messaging process, we hypothesize that patients will experience reduced depression and anxiety after the intervention, which may result in overall better patient outcomes.

## Results

Institutional review board approval was delayed due to the COVD-19 pandemic but was obtained in April 2021. Recruitment will occur from August to November 2021, and data will be analyzed in spring 2022.

## Discussion

In this paper, we describe a protocol for using text messages coupled with CBT to reduce depression and anxiety in African American patients with HF. This represents a first step in enhancing 2 intervention methods to improve the quality of life of an underrepresented group of patients with HF.

## Abbreviations

ADAPT-ITT: assessment, decision, administration, production, topical expert, integration, training, and testing
CBT: cognitive behavioral therapy
COPE: Creating Opportunities for Personal Empowerment
HF: heart failure
PHQ-9: Patient Health Questionnaire-9
STAI: State-Trait Anxiety Inventory
TXT COPE-HF: Texting With Creating Opportunities for Personal Empowerment for Patients With Heart Failure
